# Health care workers in Pearl River Delta Area of China are not vaccinated adequately against hepatitis B: a retrospective cohort study

**DOI:** 10.1186/s12879-015-1278-0

**Published:** 2015-11-22

**Authors:** Yu-Bao Zheng, Yu-Rong Gu, Min Zhang, Ke Wang, Zhan-lian Huang, Chao-Shuang Lin, Zhi-Liang Gao

**Affiliations:** Department of Infectious Diseases, The Third Affiliated Hospital, Sun Yat-sen University, Guangzhou, China; Key Laboratory of Tropical Disease Control (Sun Yat-Sen University), Ministry of Education, Tianhe road 600# Gangding, Guangzhou City, 510630 P.R. China

**Keywords:** Vaccination, Hepatitis B, Hospital, Immunity, Occupational risk

## Abstract

**Backgrounds:**

Health-care workers’ (HCWs) exposure to bodily fluids puts them at risk of hepatitis B virus HBV infection. This study investigated HBV vaccination practices and outcomes in HCWs and assessed postvaccination seroprotection across HCWs in different departments.

**Methods:**

A survey of HCWs in a Chinese public general hospital was carried out with a retrospective cohort of 1420 hospital HCWs (458 males and 962 females). HBV vaccination status (10-μg/dose used) was investigated in the cohort from vaccination records from the period of 1988 to 2008. Blood samples were collected and tested for hepatitis B surface antigen (HBsAg) and HBV antibodies (anti-HBs).

**Results:**

The overall vaccination (complete course) and HBsAg carrier rates among HCWs were 40.42 % (574/1420) and 6.13 % (87/1420), respectively. Vaccination rates differed by department, with HCWs in internal medicine (39.5 %) and emergency (42.0 %) departments having particularly low rates. The natural infection rate was 7.53 % (107/1420) among HCWs. HCWs in the department of infectious diseases (vaccination rate, 57.8 %) had the highest rate of antibody produced by natural infection (88.2 %).

**Conclusion:**

The vaccination rate was a disappointingly low among HCWs in Pearl River Delta Area of China. HCWs working in infectious diseases departments and technicians were at particularly likely to have been infected with HBV. A concerted effort is needed to bring vaccination rates up among Chinese HCWs in *Pearl River Delta Area* of southern China.

## Background

Hepatitis B virus (HBV) infection is a major cause of acute and chronic liver disease worldwide [1]—including hepatitis, cirrhosis, and hepatocellular carcinoma—and is one of the ten most common causes of death worldwide [2]. Approximately 30 % of the world’s population have serologic evidence of prior HBV infection [3]. An estimated 400 million people are infected with HBV, including 350 million chronic cases, with at least 1 million dying each year from chronic liver disease [4]. Chronic HBV infection is endemic in Asia and Africa, with more than 75 % of the world’s chronic HBsAg carriers being of Asian or African origin [5, 6].

Vaccination of health care workers (HCWs) is particularly important given their risk of exposure to blood-borne viral infections, particularly HBV, hepatitis C virus (HCV), and HIV, and vaccine-preventable diseases [7–9]. These risks are further heightened in developing countries in endemic regions such as Africa and Asia, including China [10]. Although effective anti-HBV vaccines have been available for over 10 years, many HCWs remain non-immunized against HBV [11]. Indeed HCWs, including those at particular risk of HBV infection, have been reported to have inadequate participation in vaccination programs [12, 13]. Although HBV vaccination rates are not satisfactory among HCWs in many countries, there was a complete HBV vaccination coverage among Polish surgical nurses [14].

However, currently, little is known about Chinese HCWs knowledge of HBV risk. Therefore, the aim of the present study was to assess HCWs’ HBV infection, vaccination, and seroprotection rates. The specific objectives of this study were: (1) to determine the vaccination and anti-HBV antibody statuses of HCWs in the Liver Disease Center of Pearl River Delta Area of China in a public teaching general hospital; (2) to compare vaccination seroprotection across hospital departments; and (3) to assess the HBV infection rate among Pearl River Delta Area Chinese HCWs.

## Methods

### Study design and ethics statement

The present study consisted of the retrospective analysis of medical records (1988–2008), administration of a questionnaire, and analysis of immunological blood tests. The protocol was approved by the local medical ethics committee of Third Affiliated Hospital, Sun Yat-Sen University The study was performed according to the World Medical Association Declaration of Helsinki, and. Written informed consent was obtained from all individuals included in this study. Data were entered in duplicate into a computerized database and were analyzed anonymously.

### Study population

1420 HCWs (458 males and 962 females) of our retrospective study were assembled by recruiting HCWs from *the health examination center database* (est. 1988). And 1420HCWs were recruited from the Liver Disease Center in *Pearl River Delta Area* of China (820HCWs), the People’s Hospital of Tianhe District, GuangZhou (390HCWs) and the TianHe Maternal and Child Health Hospital (210HCWs), respectively. All individuals whose data are in the database have provided written informed consent for future research and analysis of their data; nevertheless, all included subjects also provided written informed consent to participate in this study as well. All of the recruited HCWs had chosen previously to have their routine medical examinations done at the Liver Disease Center of *Pearl River Delta Area* of China in a public teaching general hospital; therefore the population is most closely representative of *Pearl River Delta Area* of China in particular. Their data were analyzed anonymously. Vaccination status was confirmed by routine medical examination reports.

**Fig. 1:**
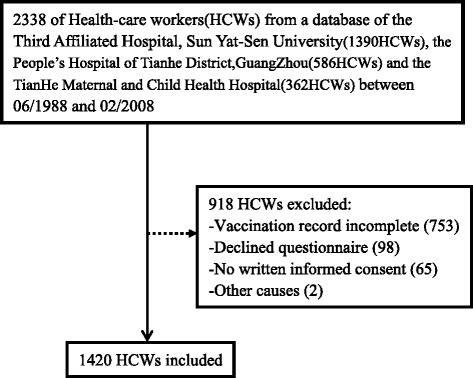
A flow diagram of study participants

The exclusion criteria were: detailed vaccination record lacking, declining to complete the survey questionnaire, and declining to provide written informed consent (see Fig. 1). The final cohort consisted of 1420HCWs had a mean age of 37.72 ± 13.37 years (only 1182 HCWs ages range, 24–51 years). By age band, there were 146 subjects ≤25 years old, 788 that were 26–39 years old, 394 that were 40–59 years old, and 92 that were ≥60 years old.

### Data collection

#### Questionnaire and medical records analysis

HCWs were asked to complete a questionnaire that asked about their vaccination history, department of employment, job category, and demographics. Prior completion of an HBV vaccination course was determined conclusively by examining the participants’ medical records.

#### Medical examination

During their 2008 routine medical examination, all HCWs in this study were subjected to testing for HBV serology, including tests for hepatitis B surface antigen (HBsAg), hepatitis surface antigen antibodies (anti-HBs), and antibody to hepatitis core antigen antibody (anti-HBc) tests. The presence of HBsAg was interpreted as indicating an active HBV infection. The presence of anti-HBs (with anti-HBc positivity) and no prior vaccination was interpreted as indicating a history of HBV infection. The presence of anti-HBs (with anti-HBc positivity) and prior vaccination was interpreted as indicating history of HBV infection in vaccinated successfully individuals. The presence of anti-HBs, but not anti-HBc, and a history of vaccination was interpreted as indicating an effective vaccination and never having been infected with HBV. The presence of anti-HBs (without anti-HBc positivity) was interpreted as indicating history of vaccinated successfully individuals.

The presence of HBsAg, anti-HBs, and anti-HBc was determined by enzyme-linked immunosorbent assay (ELISA) with kits from Shanghai Industrial Division Biotechnology Limited (China). The assays were conducted in strict accordance with the kit manufacturer’s instructions. The simple, rapid ELISA for HBsAg used is the gold-standard method for the detection of HBsAg with high sensitivity and specificity (≥99.0 %) [15]. The anti-HB titer cutoff for positive response (development of protective titers) was ≥10 mIU/mL. Data from earlier years that were originally reported with other units were converted to mIU/mL. All ELISAs were carried out in duplicate.

### Data analysis

Normality of datasets was determined using the Student’s *t*-Test. For normally distributed data, means and standard deviations (SDs) were calculated and frequency distributions across category bins were determined. For non-normally distributed data and ranges were calculated. We used the chi-square test to detect general associations. All tests were two-sided and *p* < .05 being regarded as significant. Data were analyzed using SAS statistics software.

## Results

### HBV infection status

Of the 1420 participants whose data were examined, 87 (6.13 %) were found to be positive for HBsAg and thus identified as being currently infected with HBV. As shown in Table 1, non-vaccinated HCWs were more likely to be HBsAg-positive (indicating an active infection), than were vaccinated HCWs. Table 2 revealed that of the 1420 participants, 7.53 % (107/182) had gained immunity through natural infection with HBV.

**Table 1:** Status of HBV vaccination in HCWs in Pearl River Delta Area of China (*N* = 1420)

Factor		Number	HBV vaccination status, *N* (%)		*χ* ^2^	*P*
			Vaccinated	Not vaccinated		
Sex	Male	458	140 (30.57 %)	33 (7.20 %)	0.4164	.5188
	Female	962	278 (28.90 %)	73 (7.59 %)		
Age band	<25 years	146	77 (52.7 %)	14 (7.1 %)	36.546	5.741^E-08^
	25 ~ 39 years	788	403 (51.1 %)	106 (13.5 %)		
	40 ~ 59 years	394	92 (23.4 %)	62 (15.7 %)		
	≥60 years	92	2 (2.2 %)	0 (0.0 %)		
HBsAg	Detected	87	8 (1.33 %)	79 (9.65 %)	71.583	2.659^E-17^
	Not detected	1333	593 (98.66 %)	138 (90.35 %)		
Anti-HBs level	<10 mIU/mL	644	285 (44.3 %)	75 (11.7 %)	11.772	0.00278
	1–10 mIU/mL	326	133 (40.8 %)	31 (9.5 %)		
Anti-HBc-total	Not detected	450	129 (28.7 %)	62 (13.8 %)		
	Positive	262	42 (6.99 %)	220 (26.86 %)	5.783	3.871^E-5^
	Negative	1158	559 (93.01 %)	599 (73.14 %)		
Department	Infectious diseases	106	61 (57.8 %)	17 (15.6 %)	20.660	0.0081
	General medicine	367	145 (39.5 %)	71 (19.4 %)		
	Surgery	305	186 (60.9 %)	40 (13.1 %)		
	Ob/gyn	88	56 (63.6 %)	20 (22.7 %)		
	Pediatrics	56	33 (58.9 %)	8 (14.3 %)		
	Dentistry	44	22 (50.0 %)	9 (20.5 %)		
	Emergency	88	37 (42.0 %)	6 (6.8 %)		
	Nephrology	23	13 (56.5 %)	2 (8.7 %)		
	Other	343	48 (14.0 %)	9 (2.6 %)		
Profession	Medical doctor	405	194 (47.9 %)	58 (14.3 %)	3.4387	0.4873
	Dentist	18	14 (77.8 %)	4 (22.2 %)		
	Nurse	542	311 (57.4 %)	98 (18.1 %)		
	Technician	123	42 (34.1 %)	17 (13.8 %)		
	Other	332	51 (15.4 %)	9 (2.7 %)

**Table 2:** Protective antibody status in relation to exposure route through natural infection versus vaccination by hospital department of employment and profession

Factor		Number	Portion of group with detectable protective antibodies, ratio (%)		*χ* ^2^	*P*
			Vaccinated	Infected naturally		
			*N* = 601	*N* = 107		
Department	Infectious diseases	106	42/61 (68.8 %)	15/17 (88.2 %)	1.743656	.0406
	General medicine	367	80/145 (55.2 %)	44/71 (62.0 %)	0.927921	.1767
	Surgery	305	104/186 (55.9 %)	25/40 (62.5 %)	0.695803	.2433
	Ob/gyn	88	34/56 (60.7 %)	5/20 (25.0 %)	−2.74299	.0030
	Pediatrics	56	24/33 (72.7 %)	3/8 (37.5 %)	−1.82574	.0339
	Dentistry	44	12/22 (54.5 %)	5/9 (55.6 %)	0.051297	.4795
	Emergency	88	23/37 (62.2 %)	3/6 (50.0 %)	−0.56521	.2860
	Nephrology	23	4/13 (30.8 %)	0/2 (0.0 %)	−1.09545	.1367
	Other	343	33/48 (68.8 %)	7/9 (77.7 %)	0.322184	.3737
Profession	Doctor	405	151 (77.8 %)	27 (46.6 %)	−4.68522	2^E-6^
	Dentist	18	8 (57.1 %)	1 (25.0 %)	−1.13389	.1284
	Nurse	542	192 (61.7 %)	60 (61.2 %)	−0.09085	.4638
	Technician	123	17 (40.5 %)	12 (70.6 %)	2.095346	.0181
	Other	332	31 (60.8 %)	7 (77.78 %)	0.334633	.3690
	vaccination coverage/Infected naturally rates	1420	42.32 %	7.53 %	−6.35622	2^E-7^

### Status of hepatitis B vaccination

The overall rate of vaccination (10-μg dose HBV vaccine) among HCWs was 40.42 % (574/1420). Vaccination rates were similar between males and females (Table 1). Likelihood of vaccination differed across hospital departments (Table 1). HCWs in the obstetrics and gynecology (ob/gyn) and surgery departments had the highest rates of HBV vaccination (≥60 %), whereas HCWs in the internal medicine and emergency departments had the lowest vaccination rates (≤42 %). HCWs in younger age bands, particularly the ≤25 year-old group, were more likely to have been vaccinated than HCWs than older age bands (Table 1).

### Immunity from vaccination versus natural infection

The vaccinated group was confirmed to more likely to be anti-HBs positive (i.e. protected) than the non-vaccinated group (Table 1). Of the 614 HCWs whose survey results and medical histories indicated a history of HBV vaccination, 485 (79.0 %) were found to be anti-HBs positive. The remaining 129 HCWs (21.0 %) had no protective anti-HBs despite their history of vaccination. Conversely, of the 187 HCWs who had not been vaccinated, 76 (40.6 %) were positive for anti-HBs, indicating prior exposure to the virus.

Overall, more HCWs in the present study gained seroprotection against HBV from prior infection than from vaccination. The presence of seroprotection being conferred by vaccination versus natural HBV exposure differed across departments and professions. As shown in Table 2, HCWs working in infectious diseases departments were the most likely to have gained immunity through prior exposure, as opposed to vaccination. Meanwhile, medical doctors were more likely than technicans and nurses to have gained protection through vaccination (Table 2).

## Discussion

The present study demonstrated that HCWs in southern China, overall, had a disappointingly low rate of HBV vaccination (near 40 %). Furthermore, approximately one-fifth of vaccinated HCWs lacked serological evidence of protection. Vaccination rates differed in relation to HCWs’ department of employment and profession.

HBV can be transmitted by blood transfusion, injection [16], operation, dental treatment [12], needle stick injuries, and mother-to-child vertical transmission. Estimates of the rate of HBV infection of HCWs following a one-time needle prick with an HBV-exposed needle range widely from 6 to 30 % [3]. HCWs work long hours in high-demand environments and are exposed frequently to patients’ bodily fluids, including blood, secretions, and excretions. The potential risk of acquiring blood-borne viruses through needle stick injuries has led several authors to examine the prevention of such incidents [13, 17]. Many reports have indicated that the main and, probably, only effective mechanism of preventing HBV infection among HCWs is an effective vaccine, which has been in use for about two decades [18, 19]. In 1991, the World Health Organization recommended that all countries should implement an anti-HBV vaccine in their national vaccination program by 1997 [20, 21]. Given HCWs elevated risk of HBV infection, and the related risks of infected HCWs transmitting the virus to uninfected patients, the strength of occupational protection of HCWs by vaccination should be increased aggressively [22].

The HBsAg positive rate found for HCWs in *Pearl River Delta Area* of southern China in this study was lower than expected [23]. However HCWs working in infectious diseases departments and technicians were at particularly likely to have been infected with HBV. Some portion of people who receive HBV vaccine inoculation remain at risk of HBV infection. Prior work suggests that HBV vaccination efficacy is better in younger recipients [24]. Eight vaccinated HCWs in the present study were HBsAg-positive, indicating that they were harboring an active HBV infection despite having been inoculated; presumably, they did not produce sufficient (or any) protective antibodies in response to the vaccine. Hence, serum anti-HBs levels should be assayed postvaccination to confirm whether protection has been conferred. The presence of protective antibodies in some non-vaccinated HCWs was presumably due to prior natural exposure to the virus. Nevertheless, HCWs in the vaccinated group were less likely to have a current HBV infection (indicated by HBsAg positivity) than HCWs in the non-vaccinated group, indicating that HBV vaccination had been clinically meaningful, though it was not 100 % effective.

Interestingly, the rate of workers having serologically evident protection by natural infection versus vaccination was not uniform across departments. HCWs in the infectious diseases department had the highest rate of antibody produced by natural infection; indeed more HCWs working in infectious diseases departments were infected with HBV naturally than were vaccinated. It is expected that HCWs who work in infectious diseases departments, and thus work directly with hepatitis-infected patients and samples, have a high risk of HBV exposure. Hence, the low rates of vaccination among these HCWs indicate strongly that there is a need for greater awareness of transmission risk and for promotion of HCW vaccination.

The relatively higher rate of vaccination among ob/gyn HCWs observed in this study could be related to these HCWs having gained greater awareness of the HBV vaccine as a consequence of their involvement with infant immunization plans. The four HCWs working in hemodialysis departments (encompassed in “Other” in Table 2), an area of particularly high infection risk [23], were noteworthy for characteristically obtaining anti-HBs by way of vaccination, indicating that they have a particularly high awareness of their occupational need for protection. It is noteworthy that immunity source also differed in relation to profession. Technicians with anti-HBs antibodies obtained immunity mostly through natural infection, whereas medical doctors with anti-HBs antibodies obtained immunity more often through vaccination. The low vaccination protection rate of technicians is particularly concerning given their frequent exposure to patients’ bodily fluids [25].

It is our view that HCWs who have a reasonable expectation of being exposed to blood on the job should be offered the HBV vaccine as a matter of course. Furthermore, HCWs should be informed that vaccination is not always effective and serological testing should be performed to confirm vaccine effectiveness. HCWs should be counseled regarding what steps they should take to protect their health in cases of vaccine non-responsiveness.

This study has the limitation of being largely limited to enrollment of HCWs in *Pearl River Delta Area* of southern China. So the HCWs enrolled in the present study can represent Local area of the HCWs in the *Pearl River Delta Area* of China to a certain extent. This geographical limitation limits the generalizability of these findings. Additional studies involving HCWs in other regions are needed to determine whether the patterns of data observed here are common southern China, throughout China and Asia at large, and larger retrospective studies are needed to confirm the present results. There ere also the limitation of the publication delay due to the delay in preparing the manuscript and the submission.

## Conclusion

HCWs are at an elevated risk of HBV infection due to their exposure to blood and other bodily fluids in the course of their work. Vaccination rates are disappointingly low among HCWs in southern China, with large portions of HCWs, especially HCWs working in infectious diseases departments, showing serological evidence of a natural infection history. HCWs should not only be more broadly encouraged to be vaccinated, they should be made aware that a single innoculation may not confer protection. When HCWs contract an HBV infection, they place coworkers and patients at risk of infection as well. Hence, HCWs should be vaccinated not only early, but also regularly [26]. HCWs with undetectable or low anti-HB levels in particular should receive booster vaccinations.

## Abbreviations

HCW: Health-care workers
HBV: Hepatitis B viruses
HBsAg: Hepatitis B surface antigen
anti-HBs: Antibody to hepatitis surface antigen
anti-HBc: Antibody to hepatitis core antigen
ELISA: Enzyme-linked immunosorbent assay

## References

[1] Keeffe EB, Dieterich DT, Han SH, Jacobson IM, Martin P, Schiff ER (2008). A treatment algorithm for the management of chronic hepatitis B virus infection in the United States: 2008 update. Clin Gastroenterol Hepatol.

[2] Sopipong W, Tangkijvanich P, Payungporn S, Posuwan N, Poovorawan Y (2013). The KIF1B (rs17401966) single nucleotide polymorphism is not associated with the development of HBV-related hepatocellular carcinoma in Thai patients. Asian Pac J Cancer Prev.

[3] European Association for the Study of the Liver, EASL Clinical Practice Guidelines (2012). Management of chronic hepatitis B virus infection. J Hepatol.

[4] Chen L, Zhang M, Yan Y, Miao J, Lin H, Zhang Y (2009). Sharp object injuries among health care workers in a Chinese province. AAOHN J.

[5] Hudu SA, Malik YA, Niazlin MT, Harmal NS, Sekawi Z (2014). An Overview of Hepatitis B Virus Surface Antigen Mutant in the Asia Pacific. Curr Issues Mol Biol.

[6] Adekanle O, Ndububa DA, Ayodeji OO, Paul-Odo B, Folorunso TA (2010). Sexual transmission of the hepatitis B virus among blood donors in a tertiary hospital in Nigeria. Singapore Med J.

[7] Daha TJ, Bilkert-Mooiman MA, Ballemans C, Frijstein G, Keeman JN, de Man RA (2009). Hepatitis B virus infected health care workers in The Netherlands, 2000–2008. Eur J Clin Microbiol Infect Dis.

[8] Sun ZT, Ming LH, Zhu X, Lu JH (2002). Prevention and Control of Hepatitis B in China. J Med Virol.

[9] Ramasamy P, Lintzeris N, Sutton Y, Taylor H, Day CA, Haber PS (2010). The outcome of a rapid hepatitis B vaccination programme in a methadone treatment clinic. Addiction.

[10] Fritzsche C, Becker F, Hemmer CJ, Riebold D, Klammt S, Hufert F, Akam W (2013). Hepatitis B and C: neglected diseases among health care workers in Cameroon. Trans R Soc Trop Med Hyg.

[11] Pathoumthong K, Khampanisong P, Quet F, Latthaphasavang V, Souvong V, Buisson Y (2014). Vaccination status, knowledge and awareness towards hepatitis B among students of health professions in Vientiane, Lao PDR. Vaccine.

[12] Peto TJ, Mendy ME, Lowe Y, Webb EL, Whittle HC, Hall AJ (2014). Efficacy and effectiveness of infant vaccination against chronic hepatitis B in the Gambia Hepatitis Intervention Study (1986–90) and in the nationwide immunisation program. BMC Infect Dis.

[13] Gańczak M, Szych Z (2008). Infections with HBV, HCV and HIV in patients admitted to the neurosurgical department of a teaching hospital. Neurol Neurochir Pol.

[14] Ganczak M, Ostrowski M, Szych Z, Korzeń M (2010). A complete HBV vaccination coverage among Polish surgical nurses in the light of anti-HBc prevalence: A cross-sectional sero-prevalence study. Vaccine.

[15] Kao JH (2014). Risk stratification of HBV infection in Asia-Pacific region. Clin Mol Hepatol.

[16] Hesham R, Tajunisah ME, Ilina I (2008). Risk of blood-borne infection among health care workers in two Kuala Lumpur hospitals. Med J Malaysia.

[17] Liu CJ (2014). Treatment of patients with dual hepatitis C virus and hepatitis B virus infection: resolved and unresolved issues. J Gastroenterol Hepatol.

[18] Van Herck K, Vorsters A, Van Damme P (2008). Prevention of viral hepatitis (B and C) reassessed. Best Pract Res Clin Gastroenterol.

[19] Hou JL, Liu ZH, Gu F (2005). Epidemiology and Prevention of Hepatitis B Virus Infection. International. J Med Sci.

[20] Phipps W, Honghong W, Min Y, Burgess J, Pellico L, Watkins CW (2002). Risk of medical sharps injuries among Chinese nurses. Am J Infect Control.

[21] Sangwan MCBR, Kotwal CA, Verma BAK (2011). Occupational exposure to blood and body. Med J Armed Forces India.

[22] Sarwar J, Gul N, Idris M, Anis-ur-Rehman, Farid J, Adeel MY (2008). Seroprevalence of hepatitis B and hepatitis C in health care workers in Abbottabad. J Ayub Med Coll Abbottabad.

[23] Shiv K. Sarin MD, Manoj Kumar MD. Epidemiology, Screening, and Natural History of Chronic Hepatitis B Infection. Clin Gastroenterol: Chron Viral Hepat 2010, 185–241.

[24] Okeke EN, Ladep NG, Agaba EI, Malu AO (2008). Hepatitis B vaccination status and needle stick injuries among medical students in a Nigerian university. Niger J Med.

[25] Noubiap JJ, Nansseu JR, Kengne KK, Tchokfe Ndoula S, Agyingi LA (2013). Occupational exposure to blood, hepatitis B vaccine knowledge and uptake among medical students in Cameroon. BMC Med Educ.

[26] Ali NS, Jamal K, Qureshi R (2005). Hepatitis B vaccination status and identification of risk factors for hepatitis B in health care workers. J Coll Physicians Surg Pak.

